# In vitro evaluation of antimicrobial activity of essential oils with potential application in biomaterial (castor oil based polyurethane)

**DOI:** 10.1186/1753-6561-5-S6-P43

**Published:** 2011-06-29

**Authors:** E Watanabe, CG Maia, DD Andrade, NEPECISS and Núcleo de Estudos de Prevenção e Controle de Infecção nos Serviços de Saúde (NEPECISS)

**Affiliations:** 1 Enfermagem Geral e Fundamental, Escola de Enfermagem de Ribeirão Preto – USP, Brazil; 2 Universidade de Ribeirão Preto, Ribeirão Preto, Brazil

## Introduction / objectives

Nowadays, with the multiresistant microorganisms and biofilm-related infections, diverse biomaterials with antimicrobial activity have been developed. The aim of this study was to evaluate the in vitro antimicrobial activity of essential oils with potential application in biomaterial made of the castor oil based polyurethane in this study supported by FAPESP (2010/50090-8) against reference microorganisms: *S. aureus* (ATCC 25923), MRSA (ATCC 43300), *S. epidermidis* (ATCC 14990), *E. coli* (ATCC 25922), *P. aeruginosa* (ATCC 27853) and *C. albicans* (ATCC 10231).

## Methods

The culture medium was distributed in Petri plates (20x100mm) forming a base layer of 12ml. After solidification of the medium, 8ml of medium containing microbial inoculum 1% in the range of 0.5 McFarland was distributed over the base layer to form the seed layer (seeded). On each plate were made five wells with diameters of 5mm. Aliquots of 20μl of the different essential oils were applied to each well. After the pre-incubation, the plates were incubated and the reading of the diameters of inhibition zones (mm) performed.

## Results

Essential oils with improved antimicrobial activity, in descending order, were those of the melaleuca, clove and rosemary. Moreover, the essential oils of the cedar and copaiba presented only antimicrobial activity against Gram-positive and the essential oil of the clove showed the best antimicrobial activity against *C. albicans*.

## Conclusion

In conclusion, all essential oils showed antimicrobial activity and they could be used in biomaterial to infection control, highlighting the melaleuca, clove and rosemary. In addition, *P. aeruginosa* was the most resistant microorganism and MRSA the most sensitive to essential oils.

## Disclosure of interest

None declared.

